# A Rare Case of a Solitary Central Nervous System Tuberculoma Mimicking an Intracranial Tumor

**DOI:** 10.7759/cureus.78201

**Published:** 2025-01-29

**Authors:** Aikaterini Gakidi, Eleni Faniadou, Despoina Ioannidou, Afroditi Boutou

**Affiliations:** 1 Department of Respiratory Medicine, Hippokration Hospital, Thessaloniki, GRC

**Keywords:** central nervous system tuberculosis, immunocompetent patient, intracranial pressure, pregnancy, solitary brain lesion

## Abstract

Tuberculosis (TB) can sometimes involve the central nervous system (CNS), especially among immunocompromised patients, but it is an infrequent manifestation. Among immunocompetent individuals, CNS TB has only scarcely been described in the literature; when it is manifested as a solitary lesion (tuberculoma), it can mimic other CNS tumors, appearing with clinical manifestations of increased intracranial pressure, such as headaches and vomiting. In this manuscript, we describe a case of a CNS tuberculoma, which presented as a cerebellum tumor with signs of increased intracranial pressure in an immunocompetent pregnant woman. Caesarian section and craniotomy with tumor excision were performed. Mycobacterium TB sensitive to rifampicin was identified in the tissue block, anti-TB treatment was initiated, and the patient's condition improved. The temporary immunosuppressive state of pregnancy may lead to the reactivation of TB infection, along with clinical manifestations from extrapulmonary sites, such as the CNS. Awareness of CNS tuberculomas and their clinical manifestations should be raised, as early recognition and treatment are important for a successful outcome.

## Introduction

Tuberculosis (TB) is a disease caused by the bacillus Mycobacterium tuberculosis. Although it is preventable and often curable, in 2023, TB was again the first leading cause of death worldwide from a single infectious agent, following three years in which it was replaced by coronavirus disease (COVID-19) and caused almost twice as many deaths as HIV/AIDS. According to the World Health Organization (WHO), 8.2 million infections were reported in 2023, reaching the highest peak ever since TB monitoring started in 1995. Although the burden of TB is high, following the WHO treatment guidelines, about 85% of patients can be cured [1].

TB mainly affects the lungs (pulmonary TB), but it can also involve other sites (extrapulmonary TB); this is the result of the hematogenous dissemination of the bacteria, due to either the progression of the primary infection or reactivation of previous disease, with a subsequent spread [2]. As reported by the Centers for Disease Control and Prevention (CDC), in the United States, extrapulmonary TB refers to 20.03% of TB cases; from these, approximately 1-2% involve the central nervous system (CNS) [3]. CNS TB can be attributed to several risk factors. Children, especially those suffering from malnutrition or recent measles, and patients infected by HIV are at high risk. Alcohol use disorders, neoplasms, the use of immunosuppressive agents in adults, smoking (especially among men), and diabetes mellitus are also identified as risk factors. Studies in developed countries show that most of the patients with CNS TB were foreign-born individuals [2].

CNS TB appears in three forms: tuberculous meningitis (which is most frequently encountered), intracranial tuberculoma, and spinal tuberculous arachnoiditis. CNS tuberculomas are mostly solitary lesions, but 15-34% can occur as multiple lesions as well. They can appear in the cerebrum, brain stem, cerebellum, and posterior fossa. Clinical manifestations depend on the size, number, and location of the tuberculomas and often include signs of intracranial pressure [2,4]. Such manifestations may be unusual in the West, but in India, the Near East, and parts of Asia, CNS tuberculomas represent 20-30% of all intracranial masses [4]. Intracranial tuberculomas are rarely described in the literature, and their appearance in immunocompetent patients is even less common. Our aim is to present a rare case of a CNS tuberculoma, which presented as a cerebellum tumor in an immunocompetent pregnant woman; review the pathogenic and clinical aspects of tuberculomas in pregnancy; and discuss the diagnostic and treatment challenges that arise.

## Case presentation

**Figure 1:**
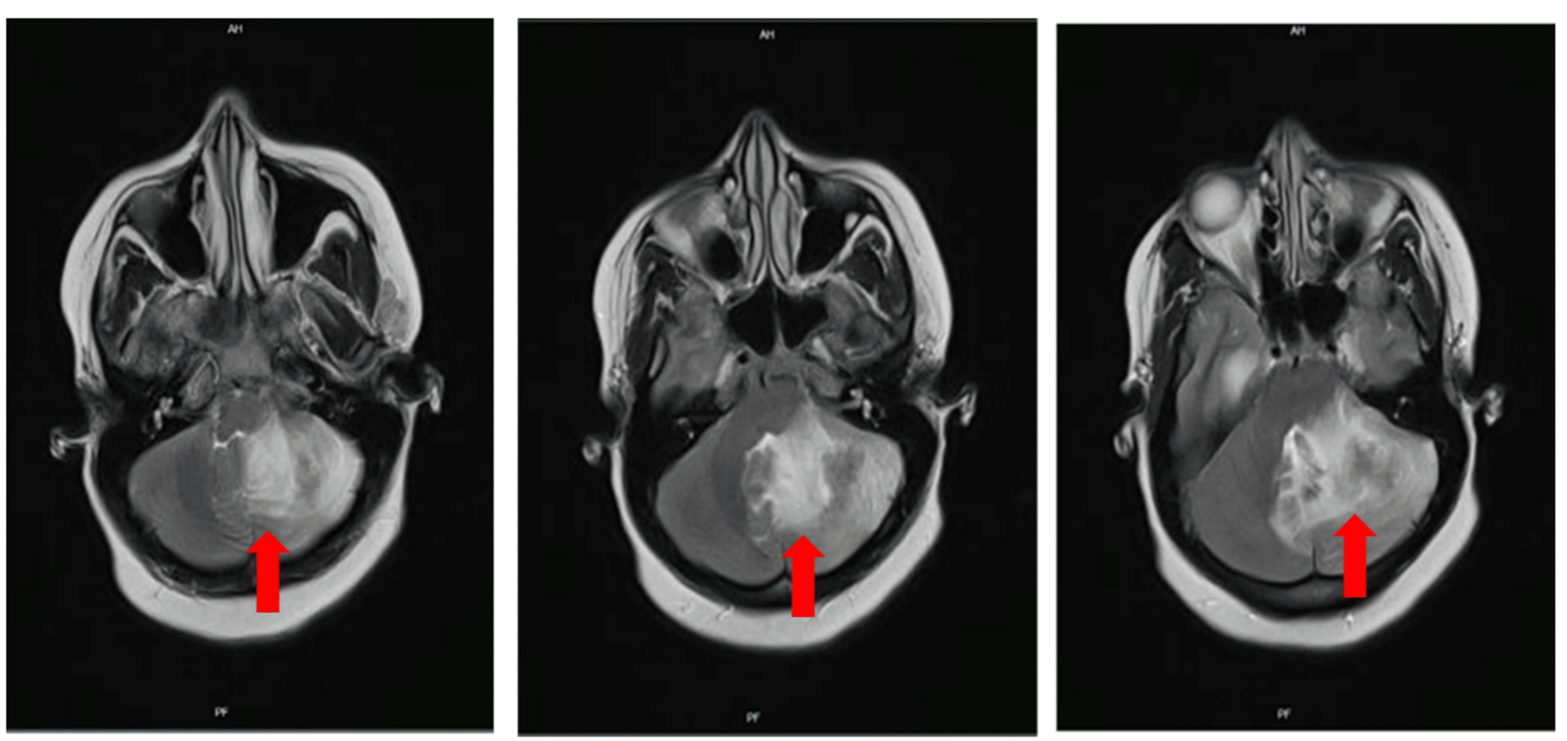
T1-weighted MRI of the brain in axial projection The red arrow marks the left cerebellar hemisphere lesion surrounded by severe perifocal edema causing mass effect and dilatation of the ventricular system.

A 20-year-old female Somalian immigrant woman at 33 weeks of pregnancy presented at a regional hospital complaining of headache. The symptom had started mildly a month ago but was worsened during the last 24 hours and was accompanied by vomiting. Brain magnetic resonance imaging (MRI) disclosed a left cerebellar hemisphere lesion surrounded by severe perifocal edema and dilatation of the ventricular system (Figure 1). Therefore, she was transferred to our hospital for neurosurgical evaluation after the placement of an external ventricular drainage catheter. Medical records of her pregnancy were not available, and she had no other medical or surgical history.

On physical examination, she was afebrile and hemodynamically stable. Respiratory, cardiovascular, and abdominal examinations were normal. She was conscious and well-oriented with a Glasgow Coma Scale (GCS) of 15 and no sign of meningeal involvement.

The patient was planned for a caesarian section with craniotomy and tumor excision at a single setting. She delivered an alive female baby weighing 2,300 g with good Apgar scores. Then, she was turned to a prone position, and a suboccipital craniotomy with left cerebellar hemispherectomy was performed. A well-circumscribed mass, which did not invade the surrounding normal tissue, was extracted and sent for pathologic examination. Postoperatively, she was treated with an antiepileptic drug (levetiracetam), low molecular weight heparin (LMWH) in a prophylactic dose, and antiedematous treatment (mannitol and dexamethasone). No further neurosurgical interventions were considered after the conduction of a brain computed tomography (CT) scan and MRI.

**Figure 2:**
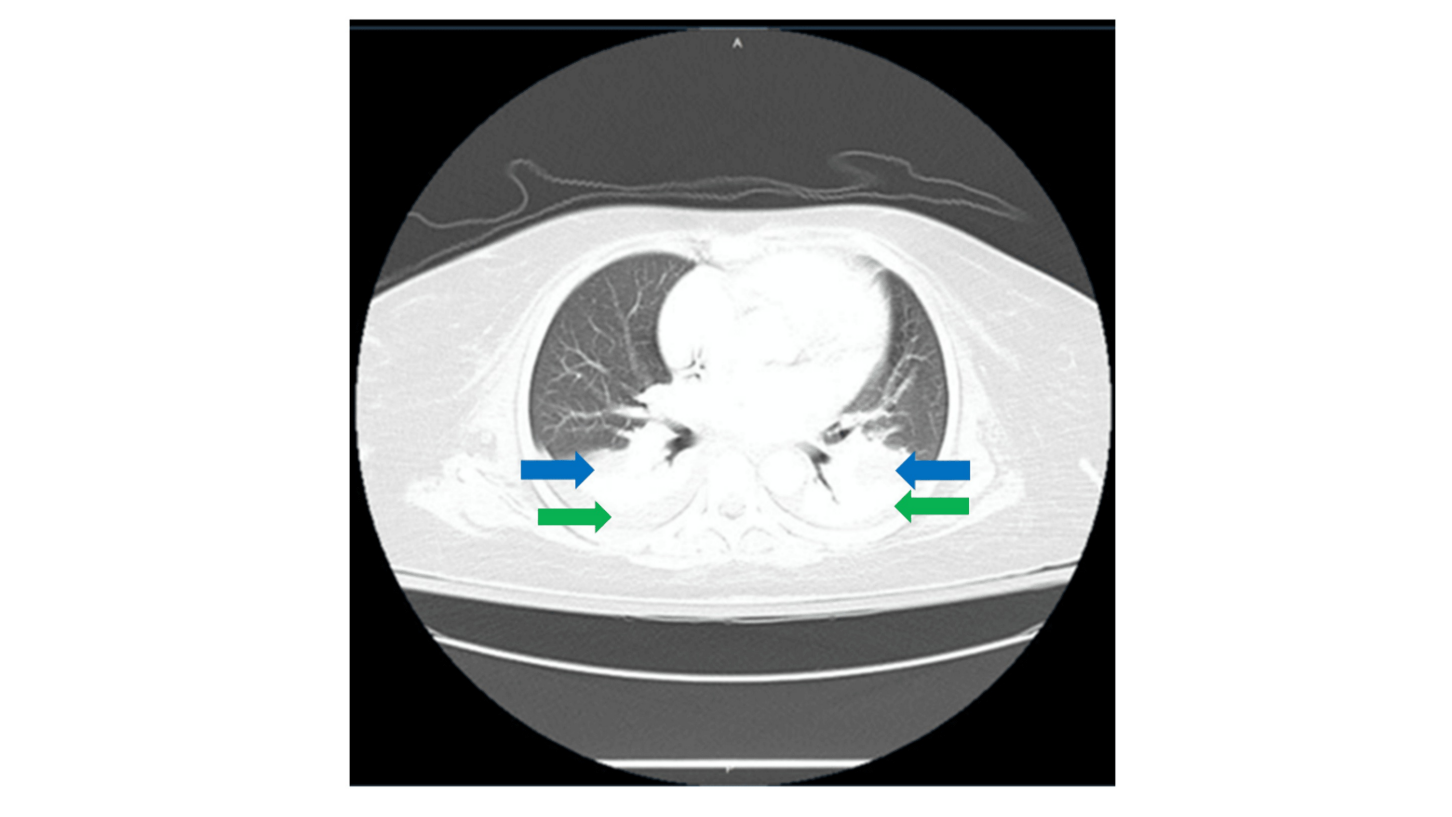
CT scan of the lower lobes in axial lung window projection The green arrows mark the bilateral pleural effusion, and the blue arrows the area of atelectasis.

The histopathologic diagnosis was necrotizing granulomatous inflammation, and the Ziehl-Neelsen stain was positive for acid‑fast bacilli. These findings were highly suggestive of tuberculoma. The Mantoux tuberculin skin test and the interferon-gamma release assay (IGRA) blood test were positive. Chest X-ray demonstrated no clear signs of active or prior pulmonary TB, whereas a chest CT scan depicted a moderate bilateral pleural effusion with atelectasis of the lower lobes (Figure 2); these findings are not TB-specific and were attributed to prior surgery. Consequently, the patient was transferred to the Pulmonary Medicine Department.

Upon further molecular testing with Xpert® M. tuberculosis/rifampin (MTB/RIF) assay, M. tuberculosis sensitive to rifampin was identified in a tissue block of the tuberculoma; this final diagnosis was confirmed within a week from the initial surgical excision of the lesion. A lumbar puncture (LP) showed clear cerebrospinal fluid (CSF) with glucose 57 mg/dL, protein 34 mg/dL, LDH 57 U/L, and five white blood cells per field of view. Xpert® assay, Ziehl-Neelsen stain, and aerobic and anaerobic culture of CSF were also negative, revealing no involvement of the meningeal system. Moreover, the lung lesions (bilateral pleural effusions and atelectases) were not TB-specific and improved quickly within a few days, so no respiratory sputum specimen was sent for mycobacterial culture or molecular testing. Due to the rareness of CNS tuberculomas in immunocompetent patients, a suspicion of human immunodeficiency virus (HIV) infection was raised. However, the subsequent serologic test proved negative and so were serologic tests for hepatitis B virus (HBV) and hepatitis C virus (HCV). Therefore, treatment with four antituberculous agents (rifampicin, isoniazid, pyrazinamide, and ethambutol) was initiated, including a high dose of rifampicin (1,200 mg/day - 20mg/kg) for a total of eight weeks; after this period, rifampicin was reduced to 600 mg/day. Isoniazid, pyrazinamide, and ethambutol doses were determined based on the patient’s body weight.

The patient was discharged after a hospitalization period of one month as gradual improvement in her clinical condition was noted. Follow-up continues until now, four months from the initial diagnosis, with an improved chest X-ray and no signs of adverse effects from the antituberculous agents.

## Discussion

In this manuscript, we present a very rare case of CNS tuberculoma located in the cerebellum of a pregnant woman. The CNS is involved in 1-2% of the total cases of extrapulmonary TB [3]. The development of a CNS tuberculoma in an immunocompetent pregnant woman is rare. On the other hand, TB may be the first manifestation of HIV infection [5]. Immunosuppression secondary to HIV infection is a risk factor for developing CNS TB [2]; however, the condition was excluded.

Pregnancy is a relatively immunosuppressive state to avoid the maternal rejection of the fetus [6-8]. During pregnancy, Th2 (e.g., IL-10) and Th3 responses (e.g., transforming growth factor b), which lead to immunosuppression, are enhanced, whereas Th1 cytokines (i.e., IL-12 and IFN-g), which cause pro-inflammatory reactions, are suppressed [6]. Maternal hormones and placental products play an important role in these immune modulations. Reactivation of Mycobacterium tuberculosis at the sites of initial infection or lymphohematogenous dissemination is inhibited by Th1 responses, and depression during pregnancy may lead to reactivation and clinical manifestations at these sites [8]. Soon after delivery, Th1 suppression is reversed rapidly. The shift from Th2 to Th1 response in the postpartum period may favor the development of disseminated lesions as well [6-8]. However, a cohort study including pregnant women in the United Kingdom between 1996 and 2008 noted that the risk of postpartum TB diagnosis was significantly increased (incidence rate ratio (IRR): 1.95; 95% CI: 1.24-3.07). No significant increase in TB diagnosis was found during pregnancy (IRR: 1.29; 95% CI: 0.82-2.03). Those results may have been affected by excessive occurrence of pregnancy complications, such as miscarriage. The significantly increased risk in the six-month postpartum period may reflect a delay between pregnancy and risk increase for TB. Diagnostic delays have been described elsewhere. Ambiguity of symptoms, frequently mimicking physiological pregnancy changes, and a conservative approach to investigations (e.g., X-rays) have been blamed for these delays. Hence, early diagnosis of peripartum TB, especially in cases like ours, is essential for the successful outcome of pregnancy.

Not only CNS tuberculomas are seldom described in the literature, but also their development in the cerebellum is unusual. As reported in some studies [9,10], the most common site for CNS tuberculomas is the cerebral hemispheres, and only a small number of them appeared in the cerebellum. There are only a few case reports of solitary CNS tuberculomas during pregnancy described in the literature [11-14], and the cerebellar is only affected in cases of multiple tuberculomas [11,15,16]. To our knowledge, our patient is the first pregnant woman with a solitary cerebellar tuberculoma.

CNS tuberculomas are hard to diagnose because of the variety of their presentation. Clinical manifestations depend on the size, number, and location of the tuberculomas and often include signs of intracranial pressure, such as headache, vomiting, vertigo, confusion, lethargy, seizures, cranial nerve palsies, or hemiparesis [2,4]. Such manifestations may be unusual in the West but in India, the Near East, and parts of Asia, CNS tuberculomas represent 20-30% of all intracranial masses [4]. Unfortunately, these lesions pose a diagnostic dilemma and are often difficult to differentiate from primary or secondary malignant neoplasms because they present with signs of elevated intracranial pressure as well [2,4]. In this case, our patient presented with headache and vomiting, and her brain MRI depicted a left cerebellar hemisphere lesion, causing a mass effect, and our first impression was a tumor. The diagnosis of CNS tuberculosis was certain only after surgical excision, histopathologic findings, IGRA, and Xpert® molecular testing, following the recommended diagnostics, in line with WHO guidelines [17]. It is important to exclude rifampicin resistance, especially when considering the fact that the patient is from Somalia, a high burden country for multidrug-resistant or rifampicin-resistant TB (MDR/RR-TB) [1].

Regarding therapeutical management, such cases of intracranial lesions during the third trimester of pregnancy with grossly increased intracranial pressure can be treated by the same setting, caesarean section, followed by excision of an intracranial lesion. Corticosteroids are used to reduce the edema [18]. It is also universally accepted that anti-TB agents are essential for the successful management of CNS TB, but there is no agreement regarding the duration of therapy. The United States (US) Centers for Disease Control and Prevention recommends 12 months of treatment for tuberculous meningitis when the M. tuberculosis strain is sensitive to all drugs. Following the CDC guidelines for sensitive TB, we initiated treatment with four anti-TB agents (rifampicin, isoniazid, pyrazinamide, and ethambutol), but we included a high dose of rifampicin (1,200 mg/day - 20 mg/kg), during the first two months of treatment [19]. Intensified treatment with a higher dose of rifampicin during the initial phase of treatment is associated with better survival outcomes, as the CSF concentration of rifampicin is lower than the plasma concentration [20].

## Conclusions

In summary, we presented a case of a cerebellar tuberculoma in an immunocompetent pregnant woman. The temporary immunosuppressive state of pregnancy may lead to the reactivation of TB infection and clinical manifestations from extrapulmonary sites, such as the CNS. Solitary intracranial tuberculomas can mimic CNS tumors appearing with clinical manifestations of intracranial pressure, such as headaches and vomiting. Given the fatal outcomes of postponed treatment, it is important to raise awareness among physicians to include CNS tuberculomas in the differential diagnosis of mass lesions in the brain, especially in pregnant women from countries with high TB incidence rates.
